# Gingival Enlargement Induced by Orthodontic Therapy: A Systematic Review

**DOI:** 10.1155/ijod/2172939

**Published:** 2026-04-15

**Authors:** María Dolores Rocha-Eiroa, Albert Ramírez-Rámiz, Lluís Brunet-Llobet, Pau Cahuana-Bartra, Judit Rabassa-Blanco, Jaume Miranda-Rius

**Affiliations:** ^1^ Department of Dentistry, Hospital Sant Joan de Déu (HSJD), University of Barcelona, Barcelona, Spain, ub.edu; ^2^ Hospital Dentistry and Periodontal Medicine Research Group, SJD Research Institute (IRSJD), Barcelona, Spain, sjdhospitalbarcelona.org; ^3^ Department of Odontostomatology, Faculty of Medicine and Health Sciences, University of Barcelona, Barcelona, Spain, ub.edu

**Keywords:** brackets, gingival enlargement, gingival overgrowth, orthodontic, systematic review

## Abstract

**Objective:**

The etiology of orthodontic‐induced gingival enlargement (OIGE) or orthodontic‐induced gingival overgrowth (OIGO) is multifactorial and is related to issues such as poor oral hygiene and the hormonal changes that are common during puberty. It is known that brackets facilitate the accumulation of dysbiotic microbial biofilm and that mechanical stress on the periodontium is a possible factor in the development of OIGE. Therefore, the objective of this systematic review was to analyze the principal factors involved in the pathogenesis of OIGE.

**Methods:**

Independent reviewers performed a systematic literature search of several databases, including PubMed, Cochrane Central Register of Controlled Trials (CENTRAL), Scopus, and Web of Science. The eligibility criteria included clinical studies assessing any type of gingival enlargement/gingival overgrowth (GE/GO) in healthy patients of any age undergoing fixed orthodontic treatment. The outcomes assessed included primary factors (mechanical stress, gingival fibrotic reaction, hormonal changes, nickel (Ni)/titanium (Ti) sensitivity, and genetics) and secondary factors (demographic variables, dysbiotic microbial biofilm, orthodontic variables, periodontal parameters, and mouth breathing). The methodological quality and risk of bias of the studies included were analyzed using the Newcastle–Ottawa Scale.

**Results:**

Titles and abstracts of 670 articles were screened. Fifty‐six articles were selected for full‐text reading. From these, 27 were included and selected for extraction. From the selected studies, 17 assessed primary factors and 12 assessed secondary factors related to GE. The results of the studies indicated that the appearance of OIGE was associated with increases in matrix metalloproteinases (MMPs) MMP‐8, MMP‐9, collagen, Ni accumulation in gingival samples, increases in estradiol and testosterone in adolescents, and genes associated with the regulation of cell proliferation and migration. The rate of OIGE was higher in males (both in children and in adolescents), and it was most frequently located in the anterior buccal regions of both arches. There was a directly proportional relationship with the duration of orthodontic treatment, probing depth (PPD), and bleeding on probing (BOP). Metal brackets, elastomeric ligatures, excess resin, and change in the polymicrobial profile favored OIGE, and thick periodontal phenotype and mouth breathing were predisposing factors for this condition. The risk of bias was “favorable” for 14‐case control, cohort, and cross‐sectional studies; medium for nine articles; and the assessment was “unfavorable” in four. The level of evidence for the studies selected was adequate, with an acceptable degree of recommendation.

**Conclusion:**

The results summarized in this systematic review suggest that the mechanical stress generated by the presence of orthodontic devices may be the main cause of the development of GE. The risk and prevalence of OIGE are highest in the adolescent population.

## 1. Introduction

Historically, gingival enlargement or gingival overgrowth (GE/GO) has been associated with the use of certain inducer drugs such as phenytoin, cyclosporine, and nifedipine [1, 2]. Currently, however, orthodontic treatment is also considered a contributing factor to GO, especially in adolescents [3–7].

The etiology of orthodontic‐induced GE (OIGE) or orthodontic‐induced GO (OIGO) is multifactorial and may be related to issues such as poor oral hygiene and the frequent hormonal changes in puberty [8]. Despite the recognition of this inflammatory factor, other possible explanations for the emergence of OIGE have also been proposed. The mechanical and chemical irritation caused by orthodontic appliances, added to the force exerted on the tooth, may favor a defensive response of the periodontium. Furthermore, prospective experimental studies have shown the allergenic effects of the nickel (Ni) present in orthodontic material on the oral mucosa and gingiva [9].

It is also known that there are certain genetically predetermined subpopulations of fibroblasts with various degrees of sensitivity to stimuli [10]. The reactive behavior of fibroblasts and the secondary inflammatory pattern have revealed a profile of cytokines and inflammatory mediators modulated by orthodontic forces that contribute to the appearance of OIGE [11–17].

Until recently, it was believed that the impact of orthodontic forces was limited to the inflammation of the periodontal ligament and the remodeling of the alveolar bone. Today, however, it is known that the mechanical stress applied to the teeth also has repercussions on the gums. Previous research has recorded increases in enzymatic biomarkers in the gingival crevicular fluid (GCF) of patients with orthodontic appliances related to mechanical stress, even when the forces are light [16–19].

The enlargement usually begins at the level of the interdental papilla, since this area of the gum has specific histological, cellular, and molecular characteristics that increase its susceptibility [20, 21].

If the overgrowth induced by orthodontic treatment is severe, it can cause resistance to tooth movement, preventing the closure of spaces in cases of diastemas or after tooth extraction, increasing the risk of recurrence and compromising periodontal health. Its practical consequences are varied and include esthetic issues, increased difficulty of oral hygiene, changes in phonetics, occlusal alterations, and, in some instances, psychological problems [6, 22].

The objective of this systematic review was to analyze the principal factors involved in the pathogenesis of OIGE/OIGO, particularly among young people.

## 2. Methods

This qualitative systematic review complied with the Preferred Reporting Items for Systematic Reviews and Meta‐Analysis (PRISMA) [23]. The protocol was registered in PROSPERO with the Reference Number CRD42022370472.

### 2.1. Eligibility Criteria

The population exposure comparison outcome (PECO) framework was used to select articles for inclusion in this systematic review. The review focused on the research question: What factors are involved in the development of OIGE or OIGO in patients with malocclusion? The four elements in the PECO framework were defined as follows: population (P), healthy patients of any age with clinical needs for fixed orthodontics; exposure (E), patients with fixed orthodontic treatment and GE; comparison (C), patients with fixed orthodontic treatment without GE; outcomes (O), factors involved in GE related to orthodontic treatment: primary factors (mechanical stress, gingival fibrotic reaction, hormonal changes, Ni/titanium (Ti) sensitivity, and genetics) and secondary factors (demographic variables, dysbiotic microbial biofilm, orthodontic variables, periodontal parameters, and mouth breathing).

#### 2.1.1. Inclusion Criteria

Clinical studies, controlled clinical trials, cohort studies, case–control studies, retrospective and prospective longitudinal observational studies, cross‐sectional and crossover studies, preferably published in English or Spanish up to December 2025.

#### 2.1.2. Exclusion Criteria

Case reports, reviews, letters, editorials, opinions, animal studies, noncomparative studies, and studies including patients with diseases that cause GE: genetic syndromes, fibromatosis, treatments with drugs that induce GE, diabetics and immunosuppressed patients, and individuals with toxic habits.

Several studies met the eligibility criteria but were not selected due to methodological limitations in the design. They were not considered to be of sufficient quality to be included in the systematic review.

### 2.2. Information Source and Search Strategy

An online literature review was conducted in Medline databases through PubMed, the Cochrane Central Register of Controlled Trials (CENTRAL), Scopus, and Web of Science up to December 2025. Other relevant studies were sought by keywords and individually.

The search was carried out using keywords, combinations of words, and MeSH terms, and an equation was written with the Boolean operators “AND” and “OR” to identify the articles to be included in the systematic review. The search equation used was “orthodontic” and “gingival enlargement” or “orthodontic” and “gingival overgrowth.”

### 2.3. Selection of Studies

The selection of articles for this systematic review was carried out in two phases. During the first phase, two independent reviewers (MDRE and ARR) separately checked the titles and abstracts of all articles identified in database searches. Articles that met the eligibility criteria were included. Full texts of references with insufficient information in the title or abstract to decide on their inclusion or exclusion were retrieved for evaluation in phase two. In this second phase, the same two examiners evaluated the full texts of the candidate articles to decide on their definitive inclusion in the systematic review. In the event of disagreement between the two examiners, the opinion of a third reviewer (PCB) was sought.

MDRE and ARR’s assessments were calibrated prior to the data extraction process. Initially, the two reviewers assessed the eligibility of studies based on a sample comprising 20%. Once they had reached an appropriate level of agreement (namely, a kappa interexaminer agreement of > 0.82), they completed the screening process for paper titles and abstracts independently. A similar calibration process was carried out for data extraction. Once they had achieved an appropriate level of agreement, they assessed the full texts of all relevant papers independently. Any disagreements were resolved via discussion, and if no consensus could be reached, a third reviewer (PCB) was consulted.

### 2.4. Data Collection

Data from the studies included were extracted and reviewed independently. Data such as authors’ names, year of publication, objectives, sample size, data collected, and study results were recorded. The GE assessment used a range of indexes, including Angelopoulos and Goaz’s apico‐coronal or vertical index, modified by the Pernu GE index (1972), the Seymour GE index (1985), the Miller and Damm GO index (1992), and the Miranda–Brunet bucco‐linguo/palatal or horizontal MB index (2001).

### 2.5. Quality Assessment (Risk of Bias)

The methodological quality and risk of bias of the studies included were analyzed using the Newcastle–Ottawa Scale for nonrandomized, cross‐sectional studies, case–control, and longitudinal cohort designs. Depending on the number of stars obtained, studies were classified into risk of bias intervals: 9–7, low risk; 6, moderate risk; <6, high risk (or half + 1 in each domain). Two reviewers (MDRE and ARR) independently performed the quality assessments, and in case of any inconsistency, the final decision was resolved by consultation and discussion with the fourth and fifth authors (LBL and JMR).

## 3. Results

### 3.1. Study Selection

A total of 670 articles were identified through database searches (165 in PubMed, 44 in Cochrane, 193 in Scopus, 235 in Web of Science, and 33 via individual subject and journal searches). After the removal of duplicates, 341 results were processed for title and abstract screening. In the first phase of the analysis, 285 articles were excluded after examining the title and abstract.

**Figure 1:**
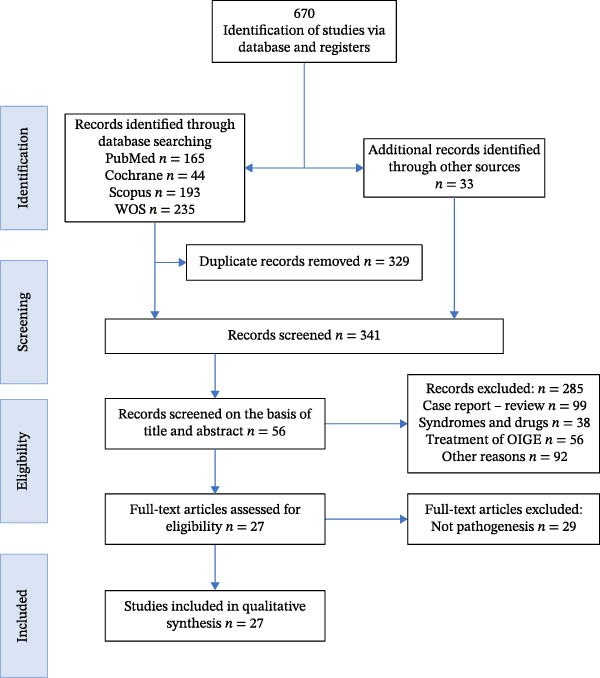
PRISMA 2020 flow diagram for new systematic reviews that included searches of databases and registers only.

Thus, in the first phase of the analysis, the sample was reduced to 56 articles. After reading the full texts, 29 further articles were excluded because they did not consider the pathogenesis of OIGE. Finally, 27 studies that met the inclusion criteria were selected for the systematic review. The identification and selection of articles are summarized in the PRISMA flowchart (Figure 1).

### 3.2. Characteristics of the Studies

Of the 27 articles included, 18 were cross‐sectional and nine were longitudinal cohort and case–control studies. The objectives in both types of study were similar, namely, to describe factors involved in GE related to orthodontic treatment. The results were divided into primary factors, that is, inducers of GE/GO due to orthodontics, and secondary factors, that is, contributors or factors associated with GE/GO also due to orthodontic treatment. Primary factors included mechanical stress, the gingival fibrotic reaction, hormonal changes, Ni/Ti sensitivity, and genetics, while secondary factors comprise demographic variables, dysbiotic microbial biofilm, orthodontic variables, periodontal parameters, and mouth breathing.

**Figure 2:**
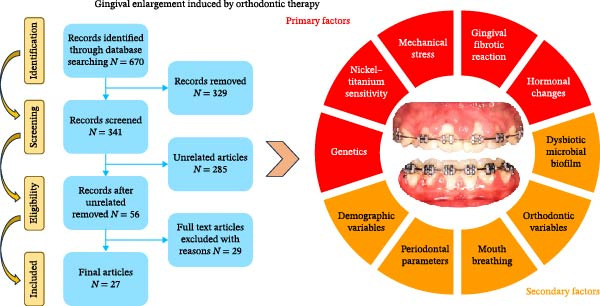
Primary and secondary factors involved in gingival enlargement related to orthodontic treatment.

The studies included in the review a) describe the histological characteristics of the interface between epithelium/connective tissues; b) analyze collagen and matrix metalloproteinases (MMPs; above all, MMP‐8 and MMP‐9) in the GCF and their relationship with mechanical stress during orthodontic treatment; and c) study levels of interleukins (ILs) and periodontal clinical parameters during the development of OIGE. Sensitization to the material used in the orthodontic appliances (Ni and Ti) was also evaluated. Other stated objectives of the cross‐sectional studies were to review the causes of OIGE in the young population analyzing hormonal values, and to assess the effect of demographic characteristics such as age and sex (Tables 1 and 2) (Figure 2).

**Table 1:** Main characteristics of primary factors studies selected.

Authors	Aim of study	Participants	Data collection	Results
Eid et al. [3]	To evaluate GE in patients of different age groups with fixed OTD	*n* = 53 G1: 21 (10–19 years) G2: 24 (20–25 years) G3: 8 (>26 years)	Clinical examination: degree of GE (0–1–2), frequency of brushing and flossing and duration of treatment	↑ GE in G1 (48%) Significant differences in frequency of GE by age group (*p* = 0.046) No significant differences in frequency of hygiene measures or duration of treatment by age group. Significant differences in GE in frequency of oral hygiene (*p* < 0.001) OH 3 times/day no GE; OH once/day degree 2 GE (28.3%). No differences in GE according to duration of treatment
Hosadurga et al. [4]	To determine the correlation between sex hormone levels and GE in patients with fixed OTD	*n* = 21 patients (16–19 years) with OTD G1: GE (12 patients) G2: no GE (9 patients)	Clinical examination: GOi, MBi, PPD, CAL, BOP, PI Hormone analysis: estradiol and progesterone in ♀, testosterone in ♂︎	GE in 57.1%; ↑PPD 75% G1/11% G2; ↑ BOP in G1 2.08 vs. G2 1.77 plaque not related to GE Estradiol/testosterone related to GE; progesterone not related to GE
Simancas‐Escorcia et al. [14]	To determine the presence and distribution of collagen III in GO with fixed OTD	*n* = 12 (>18 years) G0: 6 patients without OTD and without GO (control) G1: 6 patients with OTD and GO (cases)	Gingivectomy: gingival tissue biopsies: hematoxylin–eosin stain Masson–Goldner stain immunohistochemical analysis	Healthy individuals: stratified epithelial tissue, thin keratin layer and continuous basement membrane. Long and well‐organized collagen fibers. Collagen III adjacent to basal membrane and blood vessels Individuals with GO: keratinized hyperplastic epithelium, basal membrane with dense‐looking fibrils. Collagen fibers in dense, random bundles. Collagen III throughout the gingival connective tissue
Simancas‐Escorcia et al. [15]	Describe the histology and identify collagen I and III in gingival tissues of patients with gingival hypertrophy and OTD	*n* = 12 (>18 years) G0: 6 patients without OTD and without GO (controls) G1: 6 patients with OTD and GO (cases)	Gingivectomy: gingival tissue biopsies: Masson–Goldner staining picrosirius red/fast green staining immunohistochemical analysis	Healthy individuals: loose connective tissue, organized collagen fibers. Presence of collagen I without alterations. Slight collagen III labeling Individuals with GE: thickened epithelium. Connective tissue with collagen I ↑. Abundant collagen III networks
Ziaei et al. [24]	To investigate the potencial involvement of salivary matrix metalloproteinase (MMP)‐2 and MMP‐9 activity in orthodontic‐induced GE	*n* = 50 (10–35 years) with fixed OTD Case group: *n* = 25 with GE Case–control: *n* = 25 without GE	Clinical examination: PI, GI, PPD, CAL, GE Saliva samples: T1: 16th weeks of OTD T2: 20th weeks of OTD	MMP‐2 activity was undetectable MMP‐9 activity increased in T1 No correlation between MMP‐9 activity and GE
Simon et al. [16]	To evaluate the stress of gingival proliferative processes in OTD + VFR (vacuum form retainers)	*n* = 80 initial patients *n* = 44 (18–45 years) patients with OTD and GO	Histopathological and immunohistochemical examination: 2 mucosal samples of 2–3 mm/patient: 1st sample during treatment with fixed OTD; 2nd sample 3 months after VFR	Initial stage: ↑ presence of CD8 T lymphocytes and dendritic cells Late stage: ↑ presence of CD20 B lymphocytes; ↑stress with AWD vs. VFR; ↑stress in males compared to females
Simon et al. [17]	To evaluate gingival growth associated with fixed OTD as a result of mechanical stress and periodontal remodeling	*n* = 35 (12–38 years) patients with fixed OTD and GO	Histopathological and immunohistochemical examination: 2 mucosal samples of 2–3 mm/patient: 1st during alignment stage; 2nd during correction and finishing stages	Initial stage: ↑ presence of T lymphocytes and CD8 dendritic cells Late stage: ↑ presence of CD20 B lymphocytes
Surlin et al. [18]	To evaluate the accumulation of MMP8 in GCF at the onset of OTD as a marker of evolution of OIGE	*n* = 22 (12–29 years) patients with OTD 9 with GO: 3 with GO and PI and MI >2 6 with GO and PI and MI <2	Clinical examination: PI, MI, GO GCF samples: 1 h before, 1–4–8–24 h after and each week for 8 weeks Gingivectomy: histopathological and immunohistochemical examination	↑ MMP‐8 within 4–8 h of the insertion of OTD ↑MMP‐8 continues at maximum values in first week of GO: 3 with PI and MI >2, GO in weeks 1–3; 6 with PI and MI <2, GO in weeks 6–7 Histology: chronic inflammatory infiltrate, ↑ basal lamina ↑ intermediate layer
Surlin et al. [19]	To evaluate MMP‐9 and collagen IV in patients with GO at the start and end of OTD treatment	*n* = 45 G1: 15 OTD + GO, PI <1 and BOP 0 (9 cases) PI >1 and BOP 1 (6 cases) G2: 15 chronic GO G3: 15 healthy	Clinical examination: PI, BOP Gingivectomy: sample of gingival tissue (immunofluorescence anti‐MMP9 + anti‐ collagen IV)	↑ in the MMP‐9/collagen IV ratio, with > inflammation and fibrosis specific to GO
Orozco‐Páez et al. [25]	To demonstrate the sensitivity, reproducibility and repeatability of the blot assay for the relative quantification of MMP‐8 and MMP‐9 expression in patients with OIGE	*n* = 20 (13–35 years) G1: 5 without GO or OTD G2: 5 with GO and history of OTD G3: 5 mild GO with OTD G4: 5 moderate GO with OTD	Gingivectomy: gingival tissue biopsy: levels of MMP‐8 and MMP‐9	MMP levels: G4 > G3 > G2 G4 MMP‐8:9.9 ± 5.3 > G0; MMP‐9:12 ± 6.5 > G1 G3 MMP‐8:3.4 ± 1.2 > G0; MMP‐9:3.3 ± 0.8 > G0 G2‐G1 MMP‐8:1.2 ± 0.4 > G0, MMP‐9:1.4 ± 0.4 > G0 MMP‐8 difference between G1–G3 and G4 MMP‐9 difference between G1–G2 and G3–G4
Gursoy et al. [26]	To determine the amount of gingival Ni in OTD patients, the histological pattern of GO induced by OTD, and the dose‐dependent effect. Ni in proliferation of human keratinocytes in vitro	*n* = 10 (13–19 years patients with OTD) G1: GE sample G2: sample of 3rd molars without GE (control)	Gingivectomy: G1 tissue with GE G2 gingiva covering 3rd molars Histological analysis: atomic absorption spectrometry and keratinocyte culture with 4 concentrations of Ni (0.5, 2, 5, 10 μg)	Thickened epithelium with elongated papillae in fibrous connective tissue No differences in Ni concentration in samples with or without GE
Pazzini et al. [27]	Determine the prevalence of nickel allergy in a sample or orthodontic patients and longitudinally compare the clinical periodontal status of these individuals with that of a group of nonallergic patients	96 patients (10–43 years) *n* = 16 allergic group *n* = 16 non allergic group	Periodontal status: Loe index, clinical gingival characteristics, gingival bleeding, skin patch test (9 months after beginning) 12‐month period (1 evaluation every 3 months T1, T2, T3, T4)	17.2% Nickel allergy > prevalence in ♀︎ (94%) Differences in T3 (after 9 months) and T4 (after 12 months) between 2 groups Accumulative effect of nickel in GO
Gómez Arcila et al. [28]	Quantify the concentrations of nickel in samples of saliva, dental plaque and gingiva of individuals with fixed orthodontic appliances with and without gingival overgrowth	*n* = 24 patients with fixed OTD (20≈18−22 years‐4 > 23 years) Group A: 12 patients with GO Group B: 12 patients without GO	Gingivectomy: samples of gingival tissue, stimulated saliva and dental plaque nickel concentrations by atomic absorption spectophotometry	No statistical difference in concentrations of nickel in saliva and dental plaque Higher levels of nickel in gingiva samples in Group A (0.61 mg/L vs. 0.36 mg/L)
Orozco‐Páez et al. [29]	To determine levels of Ni and its impact on protein carbonylation in gums of patients with GO caused by OTD	*n* = 33 G1: 13 GO + OTD G2: 10 without GO with history of OTD G3: 10 without GO or OTD (control)	Gingivectomy: gingival tissue biopsies quantification of Ni protein carbonylation (western blot and mass spectrometry)	Levels of Ni in tissue G1 > G2–G3 (between 4.03 and 6.65 times higher) Protein carbonylation G1–G2 > G3 4 proteins identified
Zigante et al. [30]	Evaluate histological changes and immunohistochemical profile in patients with OTD in relation to their Ti and/or Ni allergy status	*n* = 44 (11–45 years) patient with fixed OTD 22 patients with Ti and/or Ni allergy 22 patients without Ti and/or Ni allergy	Gingivectomy: histopathological and immunohistochemical analysis	Gingiva of a patient with metal allergy: moderate fibrosis, epithelial changes, exocytosis and inflammatory infiltrates Cells sensitized to metals with ↑ in Langerhans cells and↓ in T lymphocytes: Th1 lymphocytes, Tc lymphocytes; macrophages (*p* = 0.041) and plasma cells
Baeshen et al. [31]	Examine the cellular and molecular components of gingival tissue during OTD with fixed and removable appliances	*n* = 22 G0: 8 patients without OTD (16–25 years) G1: 6 patients with fixed OTD and GE (13–17 years) G2: 8 patients with removable OTD and without GE (15–18 years)	Gingivectomy: sample of gingival tissue	CD90, CD105, CD44 no change ↑ CD24/CD146/KRT5/SOX2/NANOG/CXCL5/↓ CD133 fixed and removable ↑ ECAD/NCAD/KRT8/CXCL10/TIMP1/↓ KRT6A/MYC/MMP9 fixed ↑ MYC/MMP9/↓ CXCL10 removable KRT6A/KRT8/TIMP1 no differences between types
Simancas‐Escorcia et al. [32]	Evaluate S100A4 as a precision biomarker for personalized orthodontic risk stratification, analyzing its predictive capacity to transform orthodontic practice from reactive to predictive care paradigms	*n* = 152 (≥ 18 years) G1: healthy controls (*n* = 60) G2: orthodontic controls with GE (*n* = 31) G3: patients with established OIGE (*n* = 61)	Clinical examination: PI, GI, BOP, PPD, GE, treatment duration, appliance type Gingivectomy: gingival tissue samples	Strongest correlation with Type I collagen synthesis, treatment duration and clinical severity (*p* < 0.001) Density S100A4‐positive fibroblasts higher in G3 (*p* = 0.001) than G2 and G1 Individuals with S100A4 densities > 180 cells/mm^2^ 78% probability OIGE within 24 months Personalized orthodontic protocols and targeted preventive interventions during the critical 12–15 months therapeutic window

*Note:* Bibliographic references related to primary factors. Mechanical stress: 16, 17, 18, 19, 24, 25. Gingival fibrotic reaction: 14, 15, 16, 17, 18, 26. Ni, Ti sensitivity: 26, 27, 28, 29, 30. Hormonal changes: 3, 4. Genetics: 31, 32.

Abbreviations: AWD, arch wire devices; BOP, bleeding on probing; CAL, clinical attachment loss; GE, gingival enlargement; GI, gingival index; GO, gingival overgrowth; GOi, Miller–Damm GO index; MBi, Miranda–Brunet GO index; MI, Muhlemann bleeding index; Ni, nickel; OH, oral hygiene; OTD, orthodontic treatment; PI, Silness–Löe plaque index; PPD, probing pocket depth; Ti, titanium.

**Table 2:** Main characteristics of secondary factors studies selected.

Authors	Aim of study	Participants	Data collection	Results
Eid et al. [3]	To evaluate GE in patients of different age groups with fixed OTD	*n* = 53 G1: 21 (10–19 years) G2: 24 (20–25 years) G3: 8 (>26 years)	Clinical examination: degree of GE (0–1–2), frequency of brushing and flossing and duration of treatment	↑ GE in G1 (48%) significant differences in frequency of GE by age group (*p* = 0.046) No significant differences in frequency of hygiene measures or duration of treatment by age group. Significant differences in GE in frequency of oral hygiene (*p* < 0.001) OH 3 times/day no GE; OH once/day degree 2 GE (28.3%). No differences in GE according to duration of treatment
Hosadurga et al. [4]	To determine the correlation between sex hormone levels and GE in patients with fixed OTD	*n* = 21 patients (16–19 years) with OTD G1: GE (12 patients) G2: no GE (9 patients)	Clinical examination: GOi, MBi, PPD, CAL, BOP, PI Hormone analysis: estradiol and progesterone in ♀, testosterone in ♂︎	GE in 57.1%; ↑PPD 75% G1/11% G2; ↑ BOP in G1 2.08 vs. G2 1.77 plaque not related to GE Estradiol/testosterone related to GE; progesterone not related to GE
Pinto et al. [5]	To assess the effects of duration of fixed OTD on GE in adolescents and young adults	*n* = 260 (10–30 years): <15years /15years 20years / >20years G0: No OTD G1: OTD 1 year– G2: OTD 2 years G3: OTD 3 years	Clinical examination: IP, GI, GEi Questionnaire: sociodemographic data + oral hygiene	Significant ↑ in PI, GI, GEi: G0 < G1 < G2‐G3 (*p* < 0.05) Nonsignificant difference between G2 and G3 Risk of GE: 20–28 times higher in patients with OTD Age and GI associated with previous GE
Almansob et al. [6]	To assess factors that cause GE in adolescents and young adults	*n* = 329 (10–30 years) G0: 137 patients without OTD (control) G1: 63 patients with OTD 4–12 months G2: 68 patients with OTD 13–24 months G3: 61 patients with OTD >24 months Age: 15 (*n* = 91); 15–20 (*n* = 82); >20 (*n* = 156) Treatment stage: alignment and leveling/closing spaces/finishing	Clinical examination: OHI‐S simplified oral hygiene index calculus; GEI ant/post, upper/lower arch	Relation between duration of treatment, sex, age, hygiene and GE ↑ GE ↑ duration of treatment G0: ↑ GE lower arch, G1‐G2‐G3: ↑ GE upper arch G0‐G1‐G2‐G3: ↑ GE anteroinferior segment ↑ GE in <15years and ↓ GE in >20years in G1 and G3 ↑ GE ♂︎ in G1 and G2; ↑ GE in G3 with conventional brackets ↑GE with worse OHI‐S No relation between GE and malocclusion angle, overjet, overbite, treatment stage, and extraction
Hadzic et al. [7]	To evaluate periodontal changes in patients before, during and after fixed OTD treatment	*n* = 38 (<18 years) patients with fixed OTD	Clinical examination: PI, CI, GI, SBI, PPD, gingival recession, dental mobility Radiographic examination: OPG Start, 3 and 6 months, 1–2 years	↑ PI start to finish; ↑ CI 3–6 months; ↓ CI 1–2 years No difference CI start to finish; ↑ SBI start to finish ↑ GH 23.7% to 76.3%; ♀︎ > ♂︎. No recessions, no gingivitis, periodontitis or bone reabsorptions
Vincent‐ Bugnas et al. [33]	To assess the prevalence of OIGE during fixed OTD and related factors (biofilm)	*n* = 193 (9–30 years) patients with OTD	Clinical examination: PI, GI, PPD, gingival phenotype, papilla height (GEi) Oral hygiene questionnaire	GO in 49.7% Predisposing factors for GO: conventional metal brackets (*p* = 0.021), duration of treatment (*p* = 0.022), thick periodontal phenotype (*p* = 0.043), mouth breathing (*p* = 0.040), ♂︎(*p* = 0.035), elastomeric ligatures (*p* = 0.007), plaque (*p* = 0.004). No relationship with general health, age, ethnicity, or brushing frequency
Giannopoulou et al. [34]	To evaluate the clinical and biochemical characteristics of the periodontal inflammation around fixed OTD appliances in adolescents and adults	*n* = 80 Group A: 56 (8–16 years) without OTD Group B: 24 (10–20 years) with OTD	Clinical examination: PI, PPD, BOP, GO GCF sample: IL‐1β, IL‐8 and IL‐4	Group B: > PPD, BOP and GO Group B: > IL‐1β and IL‐8 compared with Group A IL‐4 without differences; IL‐1β, IL‐8 and IL‐4 without differences between sexes. Relation IL‐1β and IL‐8 with GO
Zanatta et al. [35]	To investigate the association between GE, periodontal status and socioeconomic characteristics in patients with fixed OTD	*n* = 330 (14–30 years) patients with OTD	Clinical examination: PI, GI, PPD, BOP, CAL, GE, excess resin Socioeconomic and demographic study. Oral hygiene questionnaire	AGE 0.69; IP 47.38%; BOP 58.72%. ↑ GE with resin and gingival bleeding No association between GE and socioeconomic status or level of education
Zanatta et al. [36]	To evaluate the association between oral health‐related quality of life (OHRQoL), GE and gingival bleeding in subjects with fixed OTD	*n =* 330 (14–30 years) patients with OTD	Clinical examination: PI, GI, PPD, BOP, CAL, GE, excess resin Socioeconomic and demographic study. BMI, DAI, OHIP‐14	AGE 0.69, BOP 58.72% AGE increase 2.83 times in OHIP‐14 ↑ OHIP‐14 with ↑ AGE, non‐Caucasian, low income, BMI >25, DAI >30 > AGE > esthetic compromise
Sheibaninia et al. [37]	Study the relationship between the use of fixed appliances and periodontal conditions during orthodontic treatment	*n =* 60 *n =* 30 (14.73 ± 2.39) patients with fixed OTD *n =* 30 (15.14 ± 1.46) patients without fixed OTD (control)	Questionnaire: sociodemographic data periodontal status	Study group: 76.7% gingival bleeding (*p* < 0.03) 53.3% GE grado 1 o < 46.6% GE grade 2 o > no gingival recession
Gong et al. [38]	To investigate the microbiological and immunological factors (IL‐1β and TGF‐β1) related to OIGE and the clinical, microbiological and immunological effects of periodontal therapy on OIGE	*n =* 24 (12–18 years) patients with OTD G1: 12 patients with OTD and GE G2: 12 patients with OTD and without GE (control)	Clinical examination: GE, PPD, PI, BOP GCF samples: Analysis of pathogens, GE, IL‐β1 and TGF‐β1. Evaluated week 0 and week 4 after periodontal treatment	PI, PBI, PPD, GE: GE group (week 0) > control group (*p* < 0.001); GE group (week 4) vs. control group (*p* > 0.05) Presence of pathogen: start G1 > G2 (*p* < 0.001); 4 weeks G1 vs. G2 (*p* > 0.05)
Lv et al. [39]	To investigate the oral microbiome and metabolome longitudinal changes associated with orthodontic treatment‐induced gingival enlargement (OT‐GE)	*n =* 26 (10–19 years)patients with OT‐GE Case group: OT‐GE 0,GOi = 0 mm. *n =* 5 OT‐GE 1, GOi > 1 mm. *n =* 11 Control group: OT‐GH, GOi = 0 mm. *n* = 5 NOT‐GH, GOi = 0 mm. *n =* 5	Clinical examination: probe depth, BOP, GOi, TQHPI, bracket type, archwire type, ligature wire, orthodontic duration, extraction correction saliva samples: bacterial shotgun metagenomics and nontarget metabolomics analysis	OT‐GE group: higher BOP, plaque score, probing depth, GOi and ligature wire type vs. controls (*p* < 0.05) No statistically significant variations in gender, type of bracket, archwire type, orthodontic duration and tooth extraction Bacteroidetes and spirochetes (OT‐GE) correlated with BOP and GOi (*p* < 0.05) Proteobacteria (NOT‐GH) negatively correlated with BOP and GOi Citrulline underexpressed in OT‐GE1 (*p* < 0.05)

*Note:* Bibliographic references related to secondary factors. Demographic variables: 3, 4, 6, 7, 33, 34, 35, and 36. Periodontal parameters: 3, 4, 5, 6, 7, 33, 34, 35, and 37. Orthodontic variables: 5, 6, 33, and 35. Dysbiotic microbial biofilm: 34, 38, and 39. Mouth breathing: 33.

Abbreviations: AGE, anterior gingival enlargement; AWD, arch wire devices; BMI, body mass index; BOP, bleeding on probing; CAL, clinical attachment loss; CI, Silness–Löe calculus index; DAI, dental aesthetics index; GE, gingival enlargement; GEi, Seymour GE index; GH, gingival hyperplasia; GI, Löe–Silness gingival index; GO, gingival overgrowth; GOi, Miller–Damm GO index; MBi, Miranda–Brunet GO index; NOT‐GH, no orthodontic treatment for periodontal health; OHIP‐14, oral health impact profile; OPG, orthopantomography; OTD, orthodontic treatment; OT‐GE, orthodontic treatment‐induced gingival enlargement; OT‐GH, orthodontic treatment for periodontal health; PBI, papillary bleeding index; PI, Silness–Löe plaque index; PPD, probing pocket depth; SBI, Muhlemann sulcus bleeding index; TQHPI, Turesky‐modified Quigley–Hein Plaque index.

### 3.3. Primary Factors

#### 3.3.1. Mechanical Stress

The appearance of OIGE was associated with the levels of MMP‐8, MMP‐9, and collagen in the gingiva of patients undergoing orthodontic treatment. In 22 participants, all of them with good plaque control, a significant increase was observed in MMP‐8 levels in the GCF, which persisted until the onset of OIGE in some patients, in the absence of inflammation [18]. In a study of 45 patients, the same authors observed that the concentrations of MMP‐9 and type IV collagen had increased in gingival samples with chronic inflammation and in samples from patients with OIGE [19]. Other authors have also reported an increase in MMP‐9 activity in the saliva of patients with GE from the fourth month of orthodontic treatment onward, but without finding a direct correlation with the degree/severity of GE [24]. Mechanical stress and orthodontic remodeling were factors associated with this increase in MMP‐8 and ‐9 and, to a variable degree, with inflammation and tissue fibrosis [18, 19, 25]. From the clinical point of view, significantly higher levels of stress were generated by arch wire devices than by vacuum‐formed orthodontic retainers [16, 17].

#### 3.3.2. Gingival Fibrotic Reactions

The histopathological description of OIGE (provided by six of the 27 studies selected) focuses on collagen, the thickness of the epithelium, and the detection of metalloproteinases. The morphological findings reported in the literature to date are confirmed: a parakeratinized acanthotic epithelium, connective tissue rich in thick collagen fibers with a chronic lymphoplasmacytic inflammatory infiltrate, and rete pegs at the border of both tissues [14–17, 26]. The distribution of collagen I and III in the extracellular matrix (ECM) was analyzed. In a comparison of gingival samples from six OIGE patients with controls, type I collagen was observed forming fibers in disordered bundles, and type III was immunolocalized close to the basement membrane [14, 15]. Gingival mucosa presented overgrowth, while in the intermediate layers the cells were hypertrophic [18].

The inflammatory infiltrate was examined, focusing on the concentration of lymphocytes and plasma cells using immunohistochemical techniques. One study analyzed samples in patients aged between 18 and 45 with fixed orthodontic appliances over a period of 1–4 years. The proportion of CD8 T cells and dendritic cells was higher in the early stage of alignment than in the later stages of correction and finishing, while CD20 B cells showed a higher count at the end of orthodontic treatment [16]. In another study, the same authors analyzed patients between 12 and 38 years old, taking two samples per participant in the same gingival area: one at the beginning of the orthodontic treatment and another toward the end. Over three‐quarters of the patients (77%) showed changes, a rate similar to that found in the previous study, with an increase in gingival volume without obvious inflammatory signs and a high number of B lymphocytes in granulation tissue [17].

#### 3.3.3. Ni–Ti Sensitivity

Several authors have studied the depositing of Ni in the gum due to the presence of this metal in the composition of orthodontic appliances [26–30]. In the study by Orozco‐Páez et al. [29], 33 patients were distributed into three groups: G1 with OIGE, G2 without OIGE but with a history of orthodontic treatment, and G3, the control group without OIGE or any orthodontic history. Eight gingival samples were taken from each patient to examine the amount of Ni and its oxidative effect on proteins. G1 and G2 presented greater Ni accumulation and protein carbonylation than G3 [29]. The histopathological characteristics of GE corresponding to orthodontic patients sensitized to Ni and Ti were analyzed. The examination of samples revealed epithelial alterations with involvement of the basement membrane and an infiltrated band of inflammation with low levels of fibrosis [30]. Gursoy et al. [26] tested the action of various concentrations of Ni in samples of enlarged gingival tissue and observed increases in the proliferation of the epithelium with low concentrations of Ni but without significant differences when compared with the controls.

#### 3.3.4. Hormonal Changes

Studying the role of sex hormones in GE in adolescent patients undergoing orthodontic treatment, Hosadurga et al. [4] found a significant relation between GE/GO and estradiol and testosterone levels, but not between GE/GO and progesterone. Eid et al. [3] analyzed three groups of patients of varying ages undergoing orthodontic treatment and presenting GE and found the highest rate of hormonal changes in the group aged between 10 and 19 (48%).

#### 3.3.5. Genetics

Baeshen et al. [31] analyzed the surface antigens of gingival cells and their gene expressions with the use of fixed and mobile orthodontic appliances. They examined 22 samples that presented various molecules associated with the regulation of gingival cell growth. The patients wore either mobile or fixed appliances. A significant increase in adhesion proteins—ectocadherin (ECAD) and neurocadherin (NCAD)—in the epithelial‐mesenchymal transition was observed. This study demonstrated the essential roles of various genes, showing their contribution in regulating cell proliferation and migration in both the removable and fixed functional appliances. Simancas‐Escorcia et al. [32] describe a new biomarker called S100A4, which is a well‐characterized marker of activated fibroblasts that are involved in pathological tissue remodeling such as GE. They observed a significant increase in S100A4 in patients with OIGE compared with two control groups: one undergoing orthodontic treatment without GE and one consisting of healthy individuals. S100A4 is the first validated precision biomarker for OIGO/GE, achieving 95% diagnostic accuracy and demonstrating exceptional clinical utility [32].

### 3.4. Secondary Factors

#### 3.4.1. Demographic Variables

The prevalence of OIGE was calculated according to the indices listed above. The prevalence of OIGE ranged between 48% and 76% in sample populations ranging from 21 to 330 individuals. In all studies, the rate was higher in males than in females, though the differences were not always significant [4, 6, 33, 34]. Children and adolescents (10–20 years) were the age group with the highest prevalence [3, 6, 34], with clinical manifestations appearing roughly 3 months after the start of orthodontic treatment in 56.2%, a figure that rose to 76.3% at 1 year and 73.7% at 2 years [7]. Some studies did not provide information on the prevalence of GE or the index used, while others did not observe any association between ethnicity, sex, level of education, and family economic status with the emergence of OIGE [35].

Other characteristics examined included sociocultural status, degree of impairment‐discomfort, esthetic appearance (smile), phonetic alterations, and difficulty chewing [35, 36]. The OIGE was most frequently located in the anterior buccal regions of both arches [4, 6, 35].

#### 3.4.2. Periodontal Parameters

Periodontal clinical parameters record changes during the course of the orthodontic treatment. Most authors found that probing depth (PPD), plaque index (PI), bleeding on probing (BOP), and gingival index (GI) worsened in individuals with orthodontics in comparison with controls [37]. A directly proportional relationship was also observed with the duration of orthodontic treatment [5, 6, 33, 34]. The increases in PPD and BOP were significantly correlated with the start of orthodontics (in the previous 3 months) and with the degree of OIGE [4, 5, 7, 34, 35]. In a study with patients aged 10–26 years, both the gingivitis and the OIGE observed were related to the frequency of daily brushing [3]. Finally, the thick periodontal phenotype was a predisposing factor for OIGE [33].

#### 3.4.3. Orthodontic Variables

The study by Almansob et al. [6], performed with 329 participants undergoing orthodontic treatment aged from 10 to 30, examined the association between local factors and OIGE. Three groups of patients were created according to the time period: initial phase (4–12 months), intermediate phase (13–24 months), and final phase (>24 months), and they were compared to a control group. None of the groups presented significant differences according to type of malocclusion, overbite, overjet, treatment stage, type of bracket, and extractions [6]. In a population of similar age, Vincent‐Bugnas et al. [33] reported that elastomeric ligatures favored OIGE (present in 58%) compared to metal or self‐ligating brackets (present in 38.9%) and that OIGE was more common with metal brackets (53%) than with ceramic brackets (26%). Conventional metal brackets increased the risk of GE by 3.5 times, a result that was associated with the duration of orthodontic treatment [6, 33]. Pinto et al. [5] analyzed a group of 260 subjects divided into four groups to assess the effect of the duration of orthodontic treatment and presence of GE and observed that patients undergoing orthodontic treatment for 1, 2, or 3 years had a 20‐ to 28‐times higher risk of GE/GO than those who did not have orthodontic appliances. Additionally, excess resin around the brackets was associated with an increase in OIGE [35].

#### 3.4.4. Dysbiotic Microbial Biofilm

The flora examined in the gingival pockets formed by OIGE in 12 cases of patients were compared with the flora of control patients. Periodontal treatment was applied in the experimental group. Subgingival plaque samples at baseline presented *Tannerella forsythia*, *Aggregatibacter actinomycetemcomitans*, *Porphyromonas gingivalis*, *Treponema denticola, and Prevotella intermedia* in significantly higher concentrations than in controls, as well as IL‐1β and TGF‐β in the GCF. After 1 month of periodontal treatment, there were no longer significant differences in these five pathogens in the two groups, but there were significant reductions in *P. gingivalis*, *A. actinomycetemcomitans*, and *T. denticola*. IL‐1β levels fell significantly, although TGF‐β levels did not [38].

The study by Giannopoulou et al. [34] of patients between 8 and 20 years old who had worn appliances for 12 months analyzed 320 samples using enzyme‐linked immunosorbent assays (ELISAs). Because of the orthodontic appliance, the periodontal flora became pathogenic, and the concentration of pro‐inflammatory cytokines IL‐1β, IL‐4, IL‐8‐ and TGF‐β1 in the GCF increased, coinciding with the manifestation of GE. The experimental group presented significantly higher values of IL‐1β and IL‐8 than the control group, though not of IL‐4 [34].

In a further study of 26 participants, cross‐omics identified specific periodontal pathogens and metabolites that have been linked to OIGO/GE. Its pathogenesis involves functional gene‐regulated metabolite metabolism influencing periodontal pathogens. *F. nucleatum*, *N. meningitidis*, and *N. subflava* have been identified as useful indicators of OIGO/GE‐related dysbiosis. It appears that these three are associated with the metabolism of citrulline, which is implicated in the development of OIGE [39].

#### 3.4.5. Mouth Breathing

In a study of 193 patients with orthodontic appliances, Vincent‐Bugnas et al. [33] found that impaired ventilation had a significant impact on gum health. More than half of the people who were mouth breathers (53.6%) had OIGE, compared to only one‐third of those who breathed through their nose (35%); the difference was statistically significant [33].

### 3.5. Risk of Study Bias

As regards the risk of bias, the planning and design of most of the selected articles were based on the Newcastle–Ottawa Scale risk of low–medium–high quality. A low risk of bias was assigned in 14 of the 27 articles, a medium risk in nine and a high risk in four. The factors that accentuated the risk of bias, especially in the cross‐sectional studies, were the small sample size (insufficient to obtain results with the desired significance) and their lack of comparability for searching for confounding variables (Table 3). Longitudinal studies generally had a risk of bias in sample selection both at baseline and during follow‐up (Table 4).

**Table 3:** Risk of bias assessment adapted for cross‐sectional studies according to the Newcastle–Ottawa Scale.

First author	Selection				Comparability	Outcome		Risk of bias
	SR	SS	NRR	S	ACV	RA	ST	
Simancas‐Escorcia et al. [32]	★	★	★	★	★	★	★	Low
Lv et al. [39]	★	★	★	★	★	★	★	Low
Zigante et al [30]	★	★	★	★★	0	★★	★	Low
Baeshen et al. [31]	0	0	★	★★	0	★★	★	Medium
Almansob et al. [6]	★	★	0	★★	★	★	★	Low
Orozco‐Páez et al. [25]	★	0	★	★	0	★★	★	Medium
Simancas‐Escorcia et al.[15]	★	0	★	★	★	★	★	Medium
Vincent‐Bugnas et al. [33]	★	★	★	★★	★	★	★	Low
Simancas‐Escorcia et al. [14]	★	0	★	★	★	★	0	High
Pinto et al. [5]	★	★	★	★	★	★	★	Low
Hosadurga et al. [4]	★	0	0	★★	0	★★	★	Medium
Gómez Arcila et al. [28]	★★	0	★	0	0	★★	★	Medium
Zanatta et al.[35]	★	★	0	★	★	★	★	Medium
Eid et al. [3]	★	★	0	★	★	★★	0	Medium
Zanatta et al.[36]	★	0	★	★	0	★★	★	Medium
Surlin et al.[19]	★	0	★	★★	0	★★	★	Low
Giannopoulou et al. [34]	★	0	★	★★	0	★★	★	Low
Gursoy et al.[26]	0	0	0	★★	0	★★	★	High

*Note:* Newcastle–Ottawa Scale: ★, criterion met; ★★, two comparability criteria met; 0, criterion not met or not reported.

Abbreviations: ACV, analysis of confounding variable; NRR, nonresponse rate; RA, results assessment; S, screening; SR, sample representativeness; SS, sample size; ST, statistical test.

**Table 4:** Risk of bias assessment for longitudinal studies according to the Newcastle–Ottawa Scale.

Cohort studies	Selection				Comparability	Outcome			Risk of bias
	SR	SNE	E	RNP	C	RA	DF	AF	
Hadzic et al. [7]	★	★	★	★	★	★	★	★	Low
Simon et al. [16]	0	★	★	0	★	★	★	0	High
Simon et al.[17]	★	★	★	0	★	★	★	0	Medium
Sheibaninia et al. [37]	★	★	★	0	★	★	0	0	High
Surlin et al. [18]	★	★	★	★	★	★	★	★	Low
Pazzini et al. [27]	★	★	★	★	★	★	★	★	Low

Case–control studies	Selection				Comparability	Exposure			Risk of bias
	CD	RC	SC	DC	C	AE	NRR		
Ziaei et al. [24]	★	★	★	★	★	★	★		Low
Orozco‐Páez et al.[29]	★	★	★	★	★	★	★		Low
Gong et al.[38]	★	★	★	★	★	★	★		Low

*Note:* Cohort and case–control studies. Newcastle–Ottawa Scale: ★, criterion met; 0, criterion not met or not reported.

Abbreviations: AE, ascertainment of exposure; AF, adequacy of follow‐up; C, comparability as a function of design or analysis; CD, case definition; DC, definition of controls; DF, duration of follow‐up; E, exposure; NRR, nonresponse rate; RA, results assessment; RC, representativeness of the cases; RNP, result not present at beginning of study; SC, selection of controls; SNE, selection of nonexposed patients; SR, sample representativeness.

The assessment of the level of scientific evidence was based on the studies’ focus on diagnosis, prevention, and detection of OIGE. The studies presented were observational, cross‐sectional, cohort, and case–control studies. In general, they were nonrandomized, valid, and controlled, reflecting an adequate/medium level of evidence, a diverse risk of bias, and a certain heterogeneity. The strength of recommendation was reasonably positive.

## 4. Discussion

This systematic review comprising studies published up to December 2025 has focused on the factors involved in the pathogenesis of OIGE/OIGO. GE is defined as the abnormal overgrowth of gum tissue. The terminology used to describe GEs has undergone significant transformation in recent years: the definitions based on histology, morphology, and other characteristics have become obsolete, and the term ‘‘gingival enlargement” is the most widely accepted and appears to be the most accurate from a scientific perspective [40].

The periodontal variables examined have been analyzed from clinical, epidemiological, and histopathological perspectives. The local action of certain components of the materials used in orthodontic appliances, such as Ni and Ti, has also been studied. Furthermore, the exclusion criteria applied in our systematic review ruled out the possibility that comorbidities or pharmacological treatments might be related to the appearance of OIGE. The tissue stress produced by the mechanical forces exerted during tooth movement appears to be the main trigger of OIGE. In fact, OIGE could be interpreted as a defense mechanism against the injury that orthodontics produces on the periodontium, which is subsequently expressed in the form of fibrotic overgrowth of the gum.

It is an established fact that subgingival dysbiosis, in combination with the host response, is considered the primary aetiopathogenic mechanism of periodontal disease. However, in the case of OIGE/GO, as with drug‐induced GE, dysbiosis is considered a contributing factor, albeit not an essential one. This overgrowth of the gums leads to the formation of gingival pseudopockets, which makes proper oral hygiene difficult and thus promotes the growth of a subgingival dysbiotic biofilm. Furthermore, it is important to note that the unregulated overgrowth of gum tissue has the potential to cause symptoms of periodontal discomfort [2, 22].

Initially, OIGE was mainly associated with the difficulty of removing bacterial plaque retained by the appliances [7, 41, 42]. Although most authors consider that bacterial plaque, oral hygiene, and OIGE are related [3, 5, 6], other studies argue that this relation is not significant [4, 33]. For their part, several authors have reported a lower frequency of brushing and a lack of motivation to maintain correct oral hygiene in adolescents, which would be the reason for the greater presence of OIGE in this population [3].

In any case, the amount of bacterial plaque may not be such a decisive factor for the development of OIGE during orthodontic treatment as initially believed, although the appliances undoubtedly facilitate its accumulation [43]. What does appear decisive is the change in the polymicrobial profile of the biofilm when it is classified as dysbiotic in situations of persistent inflammation. In this situation, the microbial plaque may be a predictor of OIGE, favoring the inflammation associated with its development [39, 44]. Periodontal pathogens do not trigger GE by themselves; mediation of fibroblastic genetics and other systemic factors of the host is necessary [4].

The increased presence of gingival bleeding has been associated with hormonal changes in adolescents undergoing orthodontic therapy. It appears that terminal vascularity may increase with a change in the microbial flora, as observed in a population of orthodontic patients aged between 13 and 19 years [4]. Some studies have found significant associations between OIGE and levels of estradiol and testosterone [3, 4]. In fact, during puberty there is a large‐scale secretion and influx of sex hormones into the target organs, and the inflamed gum presents an increase in hormonal receptors that favor this tissue trophism [8]. Their proliferation in the epithelium may also account for the development of OIGE [26]. Likewise, hormones stimulate periodontal ligament cells to synthesize more ECM [4]. The enlarged gingival tissue forms pseudopockets with a transient shift of the microbial flora towards a more anaerobic spectrum [3, 4, 34]. With some exceptions, for example Eid et al. [3], there seems to be a general consensus that the longer the duration of orthodontic treatment, the longer the time available for the hormonal activity and for the development of OIGE [6, 7, 28, 33].

Another important point is the cellular and molecular alteration related to GE. Some studies have observed that inflamed gingival tissue presents infiltrates of mature lymphocytes, which are able to synthesize immunoglobulins [16]. In a population of adolescents with fixed orthodontic appliances, some authors found that locally secreted cytokines IL‐1 and IL‐8, in the GCF, facilitate the establishment of chronic inflammation in the periodontium and that these inflammatory factors were significantly increased compared to controls [34, 45]. The design of the orthodontic bracket can influence the profile of the microorganisms in the biofilm, with changes in the microbial complexes, above all in the composition of GCF cytokines, IL‐1β, α, IL‐12, and tumor necrosis factor‐α (TNF‐α), thus leading to varying degrees of OIGE [46, 47].

Other authors have provided more information about the action of Ni in orthodontic appliances [9, 27, 28, 48]. The GI in orthodontic patients who are hypersensitive to this bioelement is usually higher than in patients with Ni‐free material. Those authors recorded a notable deterioration in gingival health between nine and twelve months after the start of orthodontic therapy, suggesting a cumulative effect of Ni during the course of the treatment. In addition, the deposition of Ni next to the biofilm may act as a mucosal irritant, being released into the saliva [49]. These gingival lesions coincided with the evaluation of Ni skin patches in sensitized patients with varying degrees of dermatitis [27]. Other authors have observed that Ni promotes the generation of reactive oxygen species (ROS). Its release into the gingival tissue may induce an oxidative reaction, promoting cell proliferation and increasing collagen synthesis, which would facilitate the development of GE [29]. However, some previous studies have analyzed the immune system’s tolerance capacity for contact with small amounts of Ni in orthodontic appliances. Apparently, if contact occurred sometime before the insertion of a piercing or a jewel containing Ni, Cr, Co, etcetra, tolerance was much better than if it occurred afterwards, due to the development of a state of hypersensitivity to Ni [50]. Another study did not report significant differences in the development of an allergy to Ni depending on whether patients wore an orthodontic device or a piercing [51]. So it may be that gingival reactions are the result of atopic dermatitis rather than a contact allergy [52].

Currently, research into OIGE is aimed at studying the impact of tissue mechanical stress produced by orthodontic forces, especially in patients with good oral hygiene [2]. In the GCF and in saliva, it has been proven that certain biomarkers, such as MMP‐8 and MMP‐9, show increases during tooth movement [18, 19, 24, 25, 53]. These elevated levels of collagenases express a tissue response equivalent to that which occurs in the healing processes of chronic ulcers [20, 53] and in chronic gingivitis [25]. However, the increase in these enzymes is not associated with greater collagen degradation, and so this situation might be considered a contributing factor to the appearance of OIGE [15, 19].

Csiszar et al. [20] already described some unique characteristics in the composition of the interdental papilla, and this could explain why this area of the gingiva is the most affected [21]. This nodule‐papillary growth also inherently involves the accumulation of bacterial plaque, which accentuates this condition [17, 21]. The pathogenic mechanisms comprise the presence of genetically predetermined gingival fibroblasts—responders—that are more sensitive to GO induction [2].

Several studies have reported the adverse effects of fixed orthodontic appliances on soft tissues, teeth, and saliva. During orthodontic treatment, some authors note changes in salivary flow, as well as a decrease in pH in unstimulated saliva [54]. Other conditions that produce a dehydrating effect on the gum, such as mouth breathing, lip incompetence, gummy smile, and hyposalivation/xerostomia, may be considered contributing factors to the development of OIGE [33, 54]. Finally, professional iatrogenesis associated with the cements used and/or mechanical irritation of the bands and arches inserted can also favor GO [38]. These local factors contribute to the appearance of OIGE, together with the gingival inflammation that generates the biofilm.

The periodontium as a whole maintains a close relationship with the movements of orthodontic forces, which affect not just the bone–periodontal ligament–tooth complex itself but also the gum. Although we did not find any articles that explain the mechanism from the inside out, we believe that the tissue stress produced by the mechanical forces exerted during tooth movement must be the main trigger of OIGE. However, this does not occur in all patients, and so we must continue to investigate why in some patients the gingiva defends itself against this mechanical aggression on the periodontium but not in others. These different responses may be due to the fact that the fibroblast presents a genetic heterogeneity, with various subpopulations of phenotypes that present different degrees of sensitivity to stimuli: responders, those that manifest GE, and nonresponders [2].

Conceivably, the molecular changes triggered by mechanical stimulation and observed in the periodontium of these responder patients may send signals to alert the gum and thicken and strengthen the supracrestal fiber bundles so as to provide greater stability to the tooth. We might even hypothesize that this defensive mechanism could be activated to protect the tooth, whose deeper periodontium suffers from severe aseptic inflammation and, consequently, excessive mobility, thus posing a risk for the tooth.

Based on our clinical experience, when the orthodontic device is removed, the mechanical forces end and this stimulus disappears, in most cases, the periodontal tissues normalize. In our bibliography search, we had serious difficulties in finding studies of OIGE in patients wearing orthodontic removable aligners. In fact, at our children’s hospital (Sant Joan de Déu), for reasons of cost and/or compliance, adolescents who opt for invisible aligners are still a minority.

The main limitation of this review is the risk of bias. In some cases, the manuscripts ranged widely in terms of the criteria applied in the study design or in the interpretation of results. The clinical relevance of these studies is limited due to the observational design, since the majority were cross‐sectional studies.

To overcome these limitations, it is recommended that high‐quality prospective studies be conducted, using standardized methodologies and case definitions. Such studies would provide more definitive insights into the relationship between orthodontic treatment and GE and may assist in predicting the probability and severity of OIGE/GO prior to orthodontic therapy.

## 5. Conclusions

This systematic review has made it possible to analyze most of the factors related to GE induced by fixed orthodontic therapy and to assess the risk, particularly in the adolescent population.

The fibrotic reaction of the gingiva could be considered a defensive response to this periodontal injury. Finally, gingival pseudopockets promote the growth of dysbiotic microbial biofilm, and some of the materials used in orthodontic appliances are considered sensitizing agents and produce an irritant effect on the gum. Also, the risk and prevalence of OIGE are highest in the adolescent population.

Future investigations of the OIGE are required to confirm whether mechanical stress could be the trigger and to determine a potential biomarker for the diagnosis of GE induced by orthodontic devices.

### 5.1. Implications for Clinical Practice

Clinicians must ensure the comfort and well‐being of patients at risk of GE, minimizing the negative impact of orthodontics on the periodontium.

### 5.2. Implications for Research

Periodontists, orthodontists, and manufacturers of orthodontic appliances should continue their investigations to see how we can reduce the risk of this gingival dimorphism in adolescents who undergo orthodontic treatment.

## Author Contributions

María Dolores Rocha‐Eiroa, Albert Ramírez‐Rámiz, Pau Cahuana‐Bartra, and Judit Rabassa‐Blanco conducted an extensive literature search and meticulously selected the manuscripts for review. Drafting of the article was undertaken collaboratively by María Dolores Rocha‐Eiroa, Albert Ramírez‐Rámiz, Lluís Brunet‐Llobet, and Jaume Miranda‐Rius. Substantive manuscript revisions were carried out by María Dolores Rocha‐Eiroa, Albert Ramírez‐Rámiz, Lluís Brunet‐Llobet, and Jaume Miranda‐Rius.

## Funding

The authors did not receive any financial support for the research or publication of this review article.

## Disclosure

The final manuscript was reviewed and approved by all authors.

## Ethics Statement

The authors have nothing to report.

## Consent

The authors have nothing to report.

## Conflicts of Interest

The authors declare no conflicts of interest.

## Data Availability

The data that support the findings of this study are available from the corresponding author upon reasonable request.
